# Medium-term mortality after hip fractures and COVID-19: A prospective multi-centre UK study

**DOI:** 10.1016/j.cjtee.2021.10.005

**Published:** 2021-10-29

**Authors:** Gareth Chan, Ashish Narang, Arash Aframian, Zaid Ali, Joseph Bridgeman, Alastair Carr, Laura Chapman, Henry Goodier, Catrin Morgan, Chang Park, Sarah Sexton, Kapil Sugand, Thomas Walton, Michael Wilson, Ajay Belgaumkar, Kieran Gallagher, Koushik Ghosh, Charles Gibbons, Joshua Jacob, Andrew Keightley, Zuhair Nawaz, Khaled Sarraf, Christopher Wakeling, William Kieffer, Benedict Rogers

**Affiliations:** a Brighton & Sussex University Hospitals NHS Trust, Brighton & Sussex Medical School, Falmer Campus, Brighton, UK; b Surrey & Sussex Healthcare NHS Trust, Canada Avenue, Redhill, UK; c Chelsea & Westminster Hospital NHS Foundation Trust, London, UK; d Ashford & St. Peter's Hospital NHS Foundation Trust, Chertsey, UK; e Western Sussex Hospitals NHS Foundation Trust (St. Richard's Hospital), Chichester, UK; f Poole General Hospital & the Royal Bournemouth Hospital, Poole, UK; g Imperial College Healthcare NHS Trust, London, UK; h Western Sussex Hospitals NHS Foundation Trust (Worthing Hospital), Worthing, UK; i Royal Surrey Hospital NHS Foundation Trust, Guildford, UK; j Frimley Health NHS Foundation Trust, Frimley, UK

**Keywords:** Hip fractures, Femoral fractures, COVID-19, Coronavirus, Mortality

## Abstract

**Purpose:**

The COVID-19 pandemic has caused 1.4 million deaths globally and is associated with a 3–4 times increase in 30-day mortality after a fragility hip fracture with concurrent COVID-19 infection. Typically, death from COVID-19 infection occurs between 15 and 22 days after the onset of symptoms, but this period can extend up to 8 weeks. This study aimed to assess the impact of concurrent COVID-19 infection on 120-day mortality after a fragility hip fracture.

**Methods:**

A multi-centre prospective study across 10 hospitals treating 8% of the annual burden of hip fractures in England between 1st March and 30th April, 2020 was performed. Patients whose surgical treatment was payable through the National Health Service Best Practice Tariff mechanism for “fragility hip fractures” were included in the study. Patients’ 120-day mortality was assessed relative to their peri-operative COVID-19 status. Statistical analysis was performed using SPSS version 27.

**Results:**

A total of 746 patients were included in this study, of which 87 (11.7%) were COVID-19 positive. Mortality rates at 30- and 120-day were significantly higher for COVID-19 positive patients relative to COVID-19 negative patients (*p* < 0.001). However, mortality rates between 31 and 120-day were not significantly different (*p* = 0.107), 16.1% and 9.4% respectively for COVID-19 positive and negative patients, odds ratio 1.855 (95% *CI* 0.865–3.978).

**Conclusion:**

Hip fracture patients with concurrent COVID-19 infection, provided that they are alive at day-31 after injury, have no significant difference in 120-day mortality. Despite the growing awareness and concern of “long-COVID” and its widespread prevalence, this does not appear to increase medium-term mortality rates after a hip fracture.

## Introduction

The ongoing COVID-19 global health pandemic due to the severe acute respiratory syndrome coronavirus 2(SARS-CoV-2 ) shows no signs of abating with over 80 million cases diagnosed and 1.7 million deaths globally at the time of publication.^1^ The time from initial symptom onset to mortality is between 2 and 8 weeks,^2^ with the median time being 18.5 days (IQR 15.0–22.0 days).^3^ Increasing cases across Europe indicate that a “second wave” of COVID-19 infections is underway in the region with daily new cases far exceeding the “first wave” experienced between March–April 2020.^4^ Initial trials have demonstrated promising results for a vaccination; however logistical challenges with regards to its distribution and public scepticism mean the associated mortality of COVID-19 will persist into the foreseeable future.^5^, ^6^, ^7^

International studies have reported the incidence of hip fractures with concurrent COVID-19 infection during the peak of the first wave of COVID-19 infections to be between 8.6% and 18.6%.^8^, ^9^, ^10^, ^11^, ^12^ Hip fractures are a significant global health burden, with 66,000 cases of fragility hip fractures occurring annually in the United Kingdom,^13^ and are associated not only with significant acute mortality rates but substantial socio-economic costs to the wider healthcare economy.

During the pandemic, presentations for hip fractures have persisted at similar rates to pre-pandemic levels.^9^^,^^11^^,^^14^ Patients sustaining hip fractures with concurrent COVID-19 infection have a 30-day mortality of approximately 30%–35%, compared to 5%–10% for COVID-19 negative patients.^8^, ^9^, ^10^, ^11^, ^12^ Even after adjusting for known variables, using the Nottingham hip fracture score (NHFS).^15^ COVID-19 infection remains an independent risk factor for subsequent 30-day mortality.^9^^,^^10^

One hundred and twenty-day outcomes are considered to be more representative of patients’ final rehabilitation and/or mortality status from hip fractures compared to 30-day mortality.^16^ One hundred and twenty-day mortality rates from hip fractures are approximately 2–3 times higher than 30-day mortality rates.^17^^,^^18^

## Methods

### Study design

This is a multicentre prospective cohort study of all consecutive patients presenting to 1 of 10 acute hospitals in South-East England with a fragility hip fracture between 1st March and 30th April , 2020.

### Study centres

Of the 10 included hospitals, 2 are regional level 1 major trauma centres (Royal Sussex County Hospital, Brighton, U.K. and St. Mary’s Hospital, London, U.K.) with the remaining 8 hospitals providing district general services to their local populations (St. Richard’s Hospital, Chichester U.K., Worthing Hospital, Worthing, U.K., Royal Surrey County Hospital, Guildford, U.K., East Surrey Hospital, Redhill, U.K., St. Peter’s Hospital, Chertsey, U.K., Chelsea & Westminster Hospital, London, U.K., Frimley Park Hospital, Frimley, U.K. and Poole General Hospital, Poole, U.K.). All hospitals provide consultant led and delivered 7-day a week hip fracture care as per National Institute of Clinical Excellence Guidance.^19^

Nine of the 10 hospitals’ collective experience of 30-day mortality from hip fractures in COVID-19 positive patients published by the authorship^9^ are included in this subsequent study assessing 120-day mortality in COVID-19 positive hip fracture patients. Collectively all the hospitals included in this study treat 8% of the annual burden of hip fractures in England.^13^

### Inclusion and exclusion criteria

Patients whose surgical treatment was payable through the National Health Service (NHS) Best Practice Tariff mechanism for “fragility hip fractures” were included in the study. No further exclusion criterion was applied.

### Data collection

The principal dataset used for this study is routinely collected as part of hospitals’ routine reporting to the National Hip Fracture Database (NHFD). This was augmented by the collection of additional patient characteristic factors including American Association of Anesthesiologists (ASA) and NHFSs.

Local laboratory reporting systems were interrogated to ascertain antigen polymerase chain reaction (PCR) on oropharyngeal and nasopharyngeal swab tests for COVID-19. Patients were considered to be COVID-19 positive if any swab result returned a positive outcome during the course of their admission. Patients were tested following national guidance at the time of their presentation to hospital, with tests being offered to anyone with a persistent new cough, dyspnoea, pyrexia, anosmia, hypoxia or with radiographic evidence of COVID-19 infection.^20^

Mortality of 120-day was ascertained from the review of local electronic patient records and communication with local general practices, in conjunction with copies of NHFD. Time to death was calculated from the date of admission (or presentation with hip fracture if the injury was sustained during an in-hospital fall) to the date of death.

### Statistical analysis

Patients were grouped into 2 cohorts for analyses: COVID-19 positive and COVID-19 negative. Statistical analysis was performed using SPSS version 27 (IBM, Massachusetts, United States of America).

Differences in baseline patient characteristics were analysed using Chi-squared for categorical data and Student's *t-*test for continuous data. Odd ratios for 31-120-day mortality was calculated from COVID-19 positive deaths, against deaths recorded for COVID-19 negative patients.

Kaplan-Meier curves were constructed to demonstrate overall survival to 120-day and for survival from 31 to 120 days. Statistical significance was set at 5% for this study.

### Ethical approval

No ethical approval was required for this study, as it utilised data collected as part of standard patient care in order to meet the United Kingdom's guidance on the care of hip fracture patients.^19^^,^^21^

## Results

This study aims to ascertain the impact of COVID-19 infection after hip fractures up to 120-day, especially given the time from symptom onset to mortality being between 2 to 8 weeks.^2^

A total of 746 patients presented to the study centres between 1st March and 30th April, 2020 with a hip fracture that were eligible for payment under the NHS Best Practice Tariff “fragility hip fracture” mechanism. No patient was excluded from this study. Twelve patients presented during the study period and were treated expectantly with end of life care pathways (as such did not have surgical treatment) from the point of admission and have not been included in mortality calculations due to their expected terminal prognosis.

Of the total cohort of 746 patients, 87 (11.7%) tested positive for COVID-19 on PCR swab testing during the perioperative period. There was a significantly higher proportion of the COVID-19 positive male patients (33/86, 38.4%) compared to COVID-19 negative patients (185/656, 28.0%, *p* = 0.048). Those positive for COVID-19 were also more likely to have intracapsular fractures (*p* = 0.015).

The mean age for all patients in the study cohort was 83.2 years, with COVID-19 patients being significantly older (*p* = 0.005) in addition to having higher mean NHFS and ASA scores (*p* = 0.003 and *p* < 0.001, respectively, Table 1).

**Table 1:** Demographic and mortality outcomes for COVID-19 positive and negative hip fractures.

Variables	COVID-19 negative	COVID-19 positive	Test	*p* value
Patients, *n* (%)	659 (83.3)	87 (11.7)		
Gender, *n* (%)			Chi-squared	0.048
Female	471 (72.0)	53 (61.4)		
Male	184 (28.0)	33 (38.4)		
Age (years)	83	86	*t*-test	0.005
ASA grade	3.0	3.3	*t*-test	0.003
NHFS	5.0	5.9	*t*-test	<0.001
Operative delay, *n* (%)			Chi-squared	0.002
No	392 (60.9)	68 (78.2)		
Yes	252 (39.1)	19 (21.8)		
Fracture type, *n* (%)			Chi-squared	0.015
IC	386 (59.8)	39 (45.9)		
EC	260 (40.2)	46 (54.1)		
30-day mortality, *n* (%)			Chi-squared	<0.001
No	620 (94.1)	56 (64.4)		
Yes	39 (5.9)	31 (35.6)		
120-day mortality, *n* (%)			Chi-squared	<0.001
No	562 (85.3)	47 (54.0)		
Yes	97 (14.7)	40 (46.0)		
31-120-day mortality, *n* (%)			Chi-squared	0.107
No	562 (90.6)	47 (83.9)		
Yes	58 (9.4)	9 (16.1)		

IC: intracapsular; EC: extracapsular (including subtrochanteric fractures); ASA: American Association of Anesthesiologists; NHFS: Nottingham hip fracture score.

**Fig. 1:**
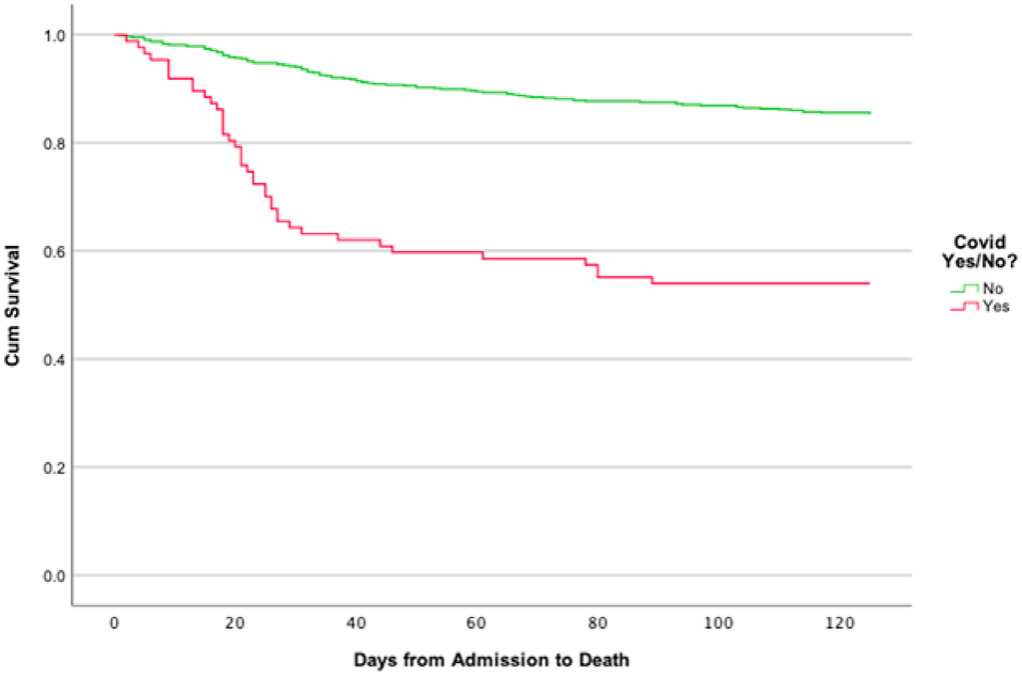
Kaplan Meier demonstrating COVID-19 positive *vs.* negative hip fracture survival from 0 to 120-days.

Overall 30-day mortality was significantly higher for COVID-19 positive patients (35.6%, 31/87) compared to COVID-19 negative patients (6.3%, 39/659, *p* < 0.001). At 120-day 46.0% (40/87) of COVID-19 positive patients had died compared to 14.7% (97/659) COVID-19 negative patients (*p* < 0.001, Fig. 1).

**Fig. 2:**
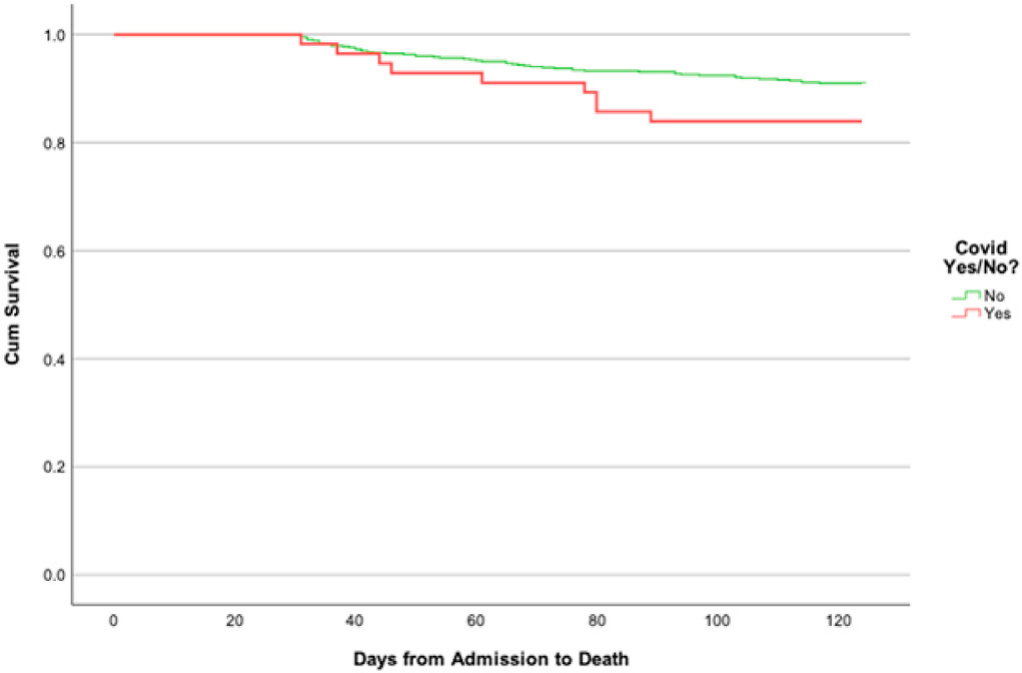
Kaplan Meier demonstrating COVID-19 positive *vs.* negative hip fracture survival from 31 to 120-days.

However, the difference in mortality rates from 31 to 120 days between COVID-19 positive (16.1%, 9/56) and COVID-19 negative patients (9.4%, 58/620) was not significantly different (*p* = 0.107, Fig. 2). The odds ratio of death between 31 and 120 days for COVID-19 positive patients was 1.855 (95% *CI* 0.865–3.978), compared to COVID-19 negative individuals (Fig. 2)

## Discussion

### *Main finding*

This study demonstrates that hip fracture patients with concurrent COVID-19 infection, provided that they are alive at day-31 after injury, have no significant difference in 120-day mortality.

### *Impact of COVID-19*

Our results have shown an overall higher 120-day mortality for COVID-19 positive patients (46.0%) than COVID-19 negative patients (14.7%, *p* < 0.001); however when analysing mortality rates between 31 and 120 days, mortality rates were 16.1% for COVID-19 positive patients and 9.4% for COVID-19 negative (*p* = 0.107). This finding is further supported by the 95% *CI* of the relative risk of death between 31 and 120-day crossing 1.0 (0.865–3.978), indicating no significant difference between the cohorts of patients.

The median time to death after the onset of COVID-19 symptoms has been reported to be 18.5 days,^3^ with the incubation period prior to symptom onset typically being 5–6 days; however this can last up to 14 days.^22^ This would encompass the majority of COVID-19 positive hip fracture patients dying within 30-day of their injury, on the assumption that they were admitted during the infected asymptomatic or symptomatic phases of the disease process.

There is increasing awareness and focus of healthcare resources on “long COVID”; an umbrella term for patients with persistent symptoms of the acute phase beyond the expected clinical duration, or those patients with lasting sequelae of their initial COVID-19 infection.^23^ Carfi et al.^24^ reported that only 12.6% of their cohort with a mean age of 56 years were symptom free 60-day after the first onset of symptoms, with 53% exhibiting symptoms of fatigue and 43% dyspnoea. Femoral neck fractures typically occur in a frail and elderly population in which up to 45% have 2 or more co-morbidities associated with increased mortality risk.^25^ Any additional impairment of physiological function in the immediate post-operative period has the potential to delay rehabilitation and long-term survival in a cohort of patients with a functional recovery rate of 30%–40%.^26^

Our results demonstrate that despite the high incidence of long COVID in the general population and its associated disabilities, this study's cohort of patients did not demonstrate increased mortality rates outside the natural course of COVID-19 mortality.

### *Patient factors*

COVID-19 positive patients were more likely to be male, older, have extra-capsular fractures and an overall greater ASA score. As compared to COVID-19 negative patients, all of 4 of which were associated with higher mortality rates.^25^^,^^27^^,^^28^ Conversely, this cohort of patients were less likely to have an operative delay (time to theatre <36 h from admission) compared to COVID-19 negative patients, which is predictive of improved short-term mortality.^29^^,^^30^ However, such differences are unlikely to account for the differences in overall 30 and 120-day mortality.

### *Impact on patient care*

During the first wave of the pandemic, presentations of hip fractures continued at pre-pandemic rates.^14^ Combined with the seasonal increase in hip fracture incidence over winter months, the burden on health services will be in-line with previous years, and likely to be greater than that of the first wave in the spring months.^31^ Comparisons between March–April 2019 and March–April 2020 have shown a reduced achievement of target times to surgery (<36 h).^9^^,^^19^ Operative delays of over 48 h is a patient-independent factor associated with increased 30-day mortality.^29^^,^^30^ It is therefore imperative with the ongoing national targets for the restoration of scheduled care within the NHS.^32^ Continued attention is paid on mitigating factors within clinicians’ control on patient mortality after hip fractures. As this study has shown, despite COVID-19 positive patients having increased short-term mortality risks, provided they survive the first 30-days after admission their medium-term mortality is no different to that of COVID-19 negative hip fracture patients.

### *Data ascertainment*

The data ascertainment for this study was 99.2% (8878/8952).

### *Strengths and limitations*

This study's cohort of 746 patients treated at the height of the first wave of the COVID-19 pandemic in the United Kingdom represents the largest series of hip fracture patients, assessing the impact of COVID-19 mortality, and is the first to assess medium-term (120-day) mortality. Additionally, the majority of hospitals involved in this study are high volume hip fracture units, collectively treating approximately 1 in every 12 hip fractures occurring in England on an annual basis.^13^ Furthermore, 2 of the study hospitals are level 1 major trauma centres, with the remaining 8 hospitals providing services to densely populated city areas with areas of high social deprivation through to sparsely populated affluent rural populations, encompassing a range of socio-economic classes that are known to affect health outcomes.^33^

The authors are unable to report on the impact of COVID-19 infection on discharge location due to the lack of robust datasets across all 10 study centres. This could potentially have demonstrated the effect of long COVID on hip fracture patients’ long-term morbidity.

### *Conclusion*

COVID-19 infection does not contribute to increased medium-term mortality rates, provided patients survive the initial acute infection period associated with significantly higher mortality rates.

## Funding

This research did not receive any specific grant from funding agencies in the public, commercial, or not-for-profit sectors.

## Ethical statement

Not required, as dataset was collected as part of routine United Kingdom hip fracture care.

## Declaration of competing interest

The authors declare that there is no conflict of interests.

## Acknowledgements

Helen Currie – Trauma Nurse Practitioner, University Hospitals Dorset NHS Foundation Trust. Joanne Brooker – Trauma & Orthopaedic Audit Officer, Brighton & Sussex University Hospitals NHS Trust, Emer Halligan – Fractured Neck of Femur Nurse Practitioner, Chelsea & Westminster Hospital NHS Foundation Trust. Siobhan Page – Advanced Trauma Nurse Practitioner, Frimley Health NHS Foundation Trust. Maria Yannopoulou – Hip Fracture Liaison Nurse, Royal Surrey Hospital NHS Foundation Trust. Rebecca Howells – Hip Fracture Liaison Nurse, Surrey & Sussex Healthcare NHS Trust. Joe Riall – Hip Fracture Data Entry Clerk, Western Sussex Hospitals NHS Foundation Trust.

## Author contributions

Gareth Chan did manuscript preparation, data analysis and statistical review. Ashish Narang did data collection and manuscript preparation. Arash Aframian, Zaid Ali, Joseph Bridgeman, Alastair Carr, Laura Chapman, Henry Goodier, Catrin Morgan, Chang Park, Sarah Sexton, Kapil Sugand, Thomas Walton, Michael Wilson, Ajay Belgaukmar, Kieran Gallagher, Koushik Ghosh, Charles Gibbons, Joshua Jacob, Andrew Keightley, Zuhair Nawaz, Khaled Sarraf, Christopher Wakeling did data collection and manuscript review. William Kieffer responsible for project conception, manuscript review and project oversight. Benedict Rogers provided project conception, manuscript review, project oversight.
